# 3D bioprinting of an intervertebral disc tissue analogue with a highly aligned annulus fibrosus via suspended layer additive manufacture

**DOI:** 10.1088/1758-5090/ad8379

**Published:** 2024-10-24

**Authors:** S R Moxon, Z McMurran, M J Kibble, M Domingos, J E Gough, S M Richardson

**Affiliations:** 1 School of Biological Sciences, University of Manchester, Manchester, United Kingdom; 2 Henry Royce Institute, University of Manchester, Manchester, United Kingdom; 3 Department of Mechanical and Aerospace Engineering, School of Engineering, Faculty of Science and Engineering, University of Manchester, Manchester, United Kingdom; 4 Department of Materials, School of Natural Sciences, University of Manchester, Manchester, United Kingdom

**Keywords:** intervertebral disc, 3D bioprinting, nucleus pulposus, annulus fibrosus, mesenchymal stem cell

## Abstract

Intervertebral disc (IVD) function is achieved through integration of its two component regions: the nucleus pulposus (NP) and the annulus fibrosus (AF). The NP is soft (0.3–5 kPa), gelatinous and populated by spherical NP cells in a polysaccharide-rich extracellular matrix (ECM). The AF is much stiffer (∼100 kPa) and contains layers of elongated AF cells in an aligned, fibrous ECM. Degeneration of the disc is a common problem with age being a major risk factor. Progression of IVD degeneration leads to chronic pain and can result in permanent disability. The development of therapeutic solutions for IVD degeneration is impaired by a lack of *in vitro* models of the disc that are capable of replicating the fundamental structure and biology of the tissue. This study aims to investigate if a newly developed suspended hydrogel bioprinting system (termed SLAM) could be employed to fabricate IVD analogues with integrated structural and compositional features similar to native tissue. Bioprinted IVD analogues were fabricated to recapitulate structural, morphological and biological components present in the native tissue. The constructs replicated key structural components of native tissue with the presence of a central, polysaccharide-rich NP surrounded by organised, aligned collagen fibres in the AF. Cell tracking, actin and matrix staining demonstrated that embedded NP and AF cells exhibited morphologies and phenotypes analogous to what is observed *in vivo* with elongated, aligned AF cells and spherical NP cells that deposited HA into the surrounding environment. Critically, it was also observed that the NP and AF regions contained a defined cellular and material interface and segregated regions of the two cell types, thus mimicking the highly regulated structure of the IVD.

## 1. Introduction

Intervertebral discs (IVDs) are fibrocartilaginous tissues which provide mechanical support to the spine and act as the main joints of the spinal column, permitting flexibility [1, 2]. The structure of IVD tissue is integral to its function and it can be broadly divided into two main regions. At the core of the IVD is an amorphous, polysaccharide-rich tissue environment called the nucleus pulposus (NP). The NP is biomechanically the softest region of the IVD (0.3–5 KPa) [3, 4] and the adult human NP is populated by rounded, chondrocyte-like NP cells. These cells are responsible for maintaining the extracellular matrix (ECM) via synthesis of new ECM macromolecules such as hyaluronan, aggrecan and type II collagen [1]. Surrounding the NP is a stiffer and more organised tissue called the annulus fibrosus (AF, ∼100 KPa) [5]. The AF is much more fibrous in nature and is comprised of a highly aligned network of primarily type I collagen fibres with proteoglycans and non-collagenous proteins present in smaller proportions. Cells within the AF adopt an elongated morphology and lie parallel to the aligned ECM [6]. Similarly to NP cells, AF cells play a pivotal role in matrix homeostasis, maintaining the composition and organisation of the extracellular environment. The two regions are integrally linked via a gradient interface called the transition zone [7]. Within this region, the AF and NP overlap and function together to aid in the absorption and distribution of loads in the spine. With ageing, there is a gradual loss of homeostatic balance between ECM synthesis and degradation [8]. While the exact mechanisms underpinning this loss have not been fully elucidated, the shift from homeostatic balance towards tissue catabolism involves changes in cell number and the activation of inflammatory pathways that result in tissue degradation and loss of structure and function of the IVD [9]. These changes may result in chronic lower back pain, with IVD degeneration representing the biggest global cause of this [10].

Research into IVD tissue development and homeostasis, as well as the combination of biological, biomechanical and microenvironmental factors that trigger degeneration is hindered by a lack of available *in vitro* models capable of reflecting the biological, biomechanical and structural anisotropy of the tissue [11]. Previous tissue engineering studies have often focussed on recreating either the NP tissue microenvironment using platforms such as polysaccharide-rich hydrogels or on recapitulating aligned AF-like structures using manufacturing processes like aligned fibre electrospinning [12–16]. A system capable of modelling both the NP and AF simultaneously remains elusive to the field. In particular, it would provide researchers with an improved *in vitro* platform for studying whole IVD biomechanics, NP-AF cell interactions, disc development and degeneration, and therapy screening whilst reducing the current reliance on animal testing models [11].

This study aims to explore if 3D bioprinting technology can be employed to fabricate a more physiologically relevant *in vitro* model of IVD tissue, by controlling the spatial deposition of cell-laden hydrogels to better mimic the complex, heterogenous nature of native tissue architectures [17]. The resolution of hydrogel bioprinting, in particular extrusion-based processes, is often limited by an ability to regulate the innate flowing behaviour of soft hydrogel materials [18, 19]. This can lead to multiple issues including structural irregularities in the final print, low printing accuracy and a lack of layer-to-layer adhesion. Consequently, this places a limit on resolution and complexity and has led to a shift towards platforms that can garner greater control over material flow and hierarchical organisation [20, 21].

One avenue of particular interest is suspended hydrogel bioprinting where the deposited, cell-laden materials are supported by an external matrix [22]. In particular, a bioprinting platform technology termed suspended layer additive manufacture (SLAM) has emerged that allows users to control the structuring and organisation of soft materials [23–25].

Here, SLAM was used to fabricate an *in vitro* tissue engineered whole IVD analogue that reflects the biological, biomechanical and structural hierarchy that is observed in native IVD tissue. We demonstrate the capacity to generate constructs designed to contain an amorphous, polysaccharide-rich NP surrounded by a stiffer, AF matrix that is reinforced with a network of aligned collagen fibres. Gellan gum polysaccharide was utilised for the NP region due to its structural similarity to hyaluronic acid which is abundant in native NP tissue while type I collagen was deposited into the AF region to reflect the collagenous nature of AF tissue [1]. The biological response of human NP and AF cells to this construct is explored with an overarching goal of creating a tissue engineering platform that can be utilised to study the biological process that underpin IVD tissue development and degeneration. Furthermore, MSC-laden constructs are generated to demonstrate the potential in utilising a bioprinted IVD for studying the role of stem cells in guiding tissue formation.

## 2. Materials & methods

### 2.1. Cell culture

Previously generated human AF and NP cell lines (clones AF58 and NP124 respectively) [26, 27] were cultured in high-glucose Dulbecco’s Modified Eagles Medium (DMEM) supplemented with 10% fetal calf serum (FCS), 100 U ml^−1^ penicillin, 100 *μ*g ml^−1^ streptomycin, 10 *μ*M ascorbate-2-phosphate and 1% sodium pyruvate (all acquired from Merck, U.K.). hTERT-immortalised human MSCs (Y201 MSCs), a kind gift from Prof Paul Genever (University of York, York, UK) that were generated via a previously published method [28], were cultured in alpha minimum essential media (*α*MEM) containing 10% FCS, 100 U ml^−1^ penicillin, 100 *μ*g ml^−1^ streptomycin, 10 *μ*M ascorbate-2-phosphate and 1% sodium pyruvate.

### 2.2. Design and SLAM bioprinting of an IVD construct

**Figure 1.**
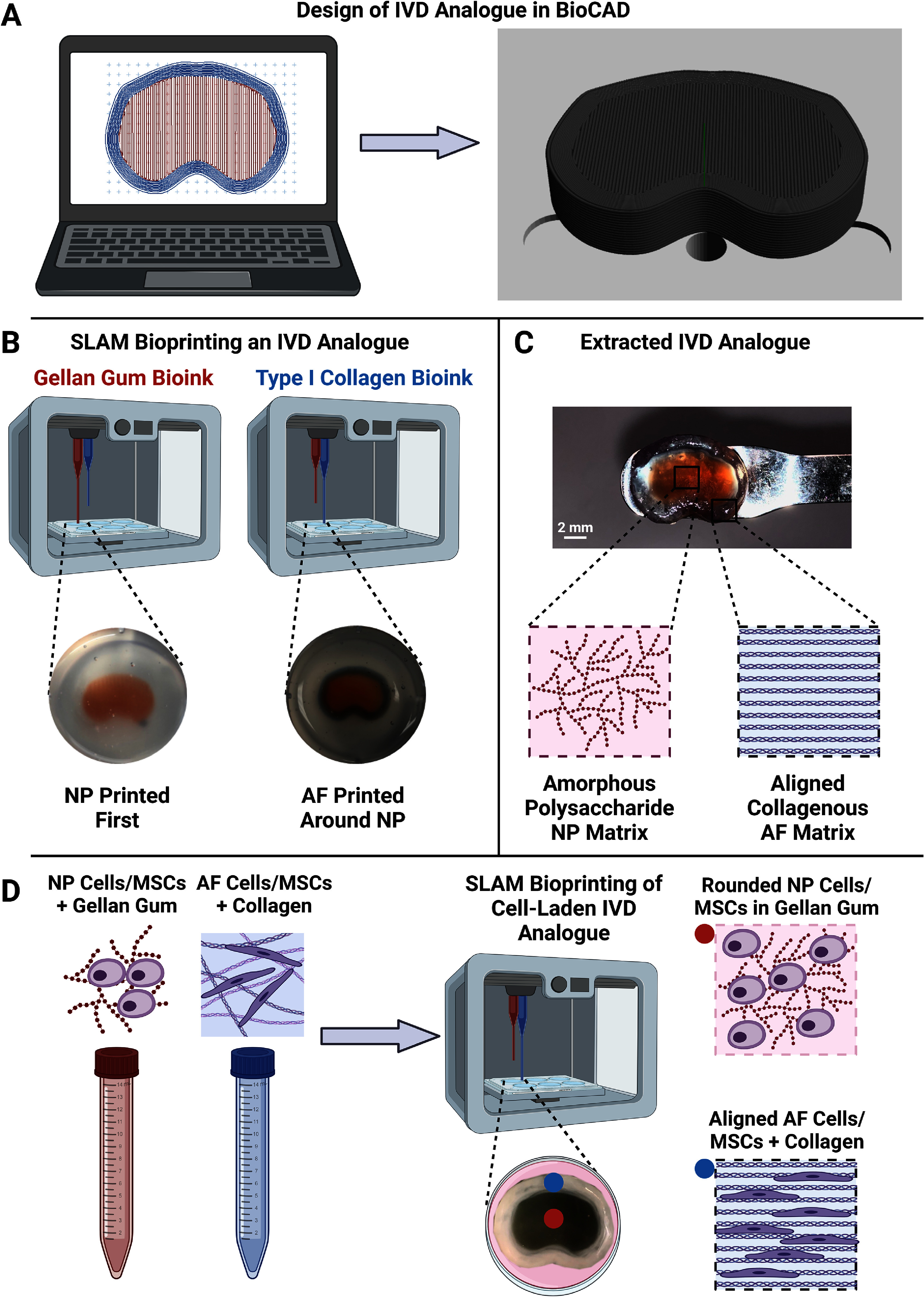
Schematic representation of the IVD bioprinting pipeline. (A) Design of an appropriate computer aided design (CAD) model. (B) SLAM bioprinting of an IVD analogue. (C) An example of a bioprinted analogue with a polysaccharide-rich core and collagenous, aligned AF. (D) Printing of a cell-laden IVD construct with embedded IVD cells or MSCs.

BioCAD™ (RegenHU, Switzerland) was used to design and generate a digital 3D model and G-code of an IVD analogue with a core NP and surrounding, annular hydrogel rings for the AF (figure 1(A)). Once generated, the G-code was opened in the corresponding software for printing using a multi-material 3D Discovery bioprinter (RegenHU, Switzerland) equipped with a microvalve inkjet printhead. The NP region was printed first followed by the AF (figure 1(B)). Both regions were extruded at a printing pressure of 20 kPa, a valve opening time of 500 ms and a dosing distance of 0.15 mm. To reflect the structural nature of the IVD, 1% gellan gum polysaccharide (prepared by dissolution in dH_2_0 at 80 °C) was used as the material for the NP region of the construct and type I collagen (Purecol^®^ EZ Gel) was utilised for the AF region of the print. Flow of the materials was controlled post-ejection from the printhead via deposition into 6-well plates containing a 0.5% agarose fluid gel bath prepared as previously reported (dissolution in dH_2_0 at 80 °C and exposed to constant shear forces through gelation) [24, 25]. Once both regions were deposited, 5 ml *α*MEM was added to the surface of the fluid gel support to trigger ionic gelation of gellan before incubation in supplemented media at 37 °C for 40 min to trigger collagen gelation. The resulting constructs were then extracted from the fluid gel support matrix (figure 1(C)) and progressed for culture or analysis.

For cell-based experiments, constructs were generated containing NP and AF cells in their respective regions, or human Y201 MSCs in both regions. Prior to bioprinting, cells were trypsinised and resuspended in the corresponding material (gellan for the NP, collagen for the AF) at a density of 2 × 10^6^ cells ml^−1^. The resulting cell-laden materials were then loaded onto the bioprinter for printing and crosslinking as described previously (figure 1(D)). Cell-laden constructs were extracted and cultured for up to 28 d (37 °C, 20% O_2_, 5% CO_2_) in high-glucose DMEM containing 1% FCS, 1X insulin-transferrin-selenium (ITS-X), 100 *μ*M ascorbate-2-phosphate, 1.25 mg ml^−1^ bovine serum albumin (BSA), 10^−7^M dexamethasone, 5.4 *μ*g ml^−1^ linoleic acid, 40 *μ*g ml^−1^ L-proline, 100 U ml^−1^ penicillin, 100 *μ*g ml^−1^ streptomycin and 0.25 *μ*g ml^−1^ amphotericin, with the addition of 10 ng ml^−1^ TGF*β*3. Media was changed every 2–3 d.

### 2.3. Electron microscopy

Printed IVD cell-laden constructs were fixed 48 h post-printing in 4% paraformaldehyde (PFA) and 2.5% glutaraldehyde in 0.1 M Hepes buffer (pH 7.2) for 1 h. Samples were then washed and stored in PBS for 24 h prior to analysis of microarchitecture using scanning electron microscopy (SEM) and transmission electron microscopy (TEM).

Samples for SEM were freeze-dried overnight, sputter coated with gold/palladium (80/20) and imaged using a FEI Quanta 200. The images were acquired under high vacuum conditions with a voltage of 15 kV and a pressure of 3.2 × 10−5 Torr.

For TEM, constructs were post-fixed with 1% osmium tetroxide and 1.5% potassium ferrocyanide in 0.1 M cacodylate buffer (pH 7.2) for 1 h and in 1% uranyl acetate in water overnight. Samples were then dehydrated in ethanol, infiltrated with TAAB’s low viscosity resin and polymerized for 24 h at 60 °C. Sections were prepared with a Reichert Ultracut ultramicrotome (Leica Microsystems, UK) and observed with a FEI Tecnai 12 Biotwin microscope (FEI™ Company, USA) at 100 kV accelerating voltage. Images were taken with a Gatan Orius SC1000 CCD camera (Gatan, UK).

### 2.4. Atomic force microscopy (AFM)

AFM was employed to study the structure of the bioprinted cellular IVD analogues both 1 day post-printing and following a 28 day culture period. Samples were extracted from culture and air dried overnight on glass microscope slides. Once dried, any excess salt and media components present in the dry samples were then removed by washing for 5 min in dH_2_O followed by a further overnight drying process at room temperature. The surface of the dried samples was probed using a Bruker Resolve AFM in tapping mode (Bruker, U.K.). Analyses were conducted on dry samples in air using a Scanasyst probe. A variety of scan sizes were performed (10–100 *μ*m) across samples in triplicate using a scanning frequency of 0.5 Hz and 512 samples per line with a total of 12 images captured per region. Scans were subsequently reconstructed in Nanoscope Analysis (Bruker, U.K.).

Mechanical profiles of the cultured bioprinted constructs were generated using AFM under the same conditions as above. Quantitative nanoscale mechanical mapping was performed in both the NP and AF regions of the construct. Nanoscope Analysis was subsequently used to derive the Derjaguin-Muller-Toporov (DMT) modulus of each region from the resulting mechanical surface maps over the duration of the culture period.

### 2.5. Cell viability assay

A live/dead assay was performed on cell-laden bioprinted constructs 24 h after printing to ensure the fabrication process did not produce any initial cytotoxic effects. Samples were washed with Dulbecco’s phosphate buffered saline (DPBS) and incubated with 3 *μ*M calcein AM (live dye) and 5 *μ*M propidium iodide (cell death dye) in DPBS for 30 min at 37 °C. Samples were then imaged using an EVOS™ digital colour fluorescence microscope (Fisher, U.K.).

### 2.6. Cell tracking

Cell tracker dyes were utilised to evaluate the spatial positioning of cells within the bioprinted constructs 24 h after printing. Human NP and AF cells were pre-labelled with CellTracker™ Green and CellTracker™ Red respectively as per manufacturer instructions (1:1000 in media, 30 min at 37 °C; ThermoFisher Scientific) prior to printing. Whole construct images were acquired using a Leica M205 stereo fluorescence microscope. Samples were then fixed for 30 min in 4% PFA and sectioned to acquire microscopic images of the tracked cells in the different regions of the construct. The constructs were embedded in 6% agarose and sliced into 100 *μ*m thick sections using a vibratome [23] before imaging using an EVOS™ digital colour fluorescent microscope. Green and red labelled cells were then pseudo-coloured as yellow and purple respectively to clarify differences between viability and cell-tracker images.

### 2.7. Cytoskeletal imaging

Phalloidin staining was employed to allow for analysis of cytoskeletal organisation and cell morphology in the two regions of the bioprinted construct. Samples were removed from culture after 28 d, washed with DPBS and fixed for 30 min in 4% paraformaldehyde (PFA). The cells within the constructs were then permeabilised in 0.2% Triton-X in PBS for 30 min. Samples were then washed 3 times in DPBS and blocked with 10% BSA for 1 h, washed 3 times with PBS and incubated with 6.6 *μ*M phalloidin Alexa Fluor 594 for 1 h. Excess phalloidin was removed with 3 further DPBS washes before incubation for 30 mins at room temperature with 4′,6-diamidino-2-phenylindole (DAPI, 1:1000 in DPBS). Samples were then embedded, sectioned and imaged as in 2.6.

### 2.8. Hyaluronic acid immunostaining

A hyaluronic acid binding protein was used to screen for hyaluronan deposition in the NP region of the bioprinted constructs. Samples were removed from culture after 28 d and fixed, washed and blocked in 10% BSA. Constructs were then incubated overnight at 4 °C with biotinylated hyaluronic acid binding protein (1:50 in 10% BSA; Amsbio, AMS.HKD-BC41), washed 3 times in DPBS and incubated for 2 h at room temperature in streptavidin Alexa Fluor 488 (1:500 in DPBS). Constructs were subsequently washed, stained with DAPI, sectioned and imaged as in 2.6.

### 2.9. Histology

After 28 d in culture, cell-laden constructs were washed in DPBS and fixed in 4% PFA for 24 h. Samples were dehydrated in 70% ethanol for 24 h and processed to paraffin wax before 7 *μ*m sections were prepared with a microtome. Sections were dewaxed and washed in dH_2_O. The sections were then stained with a combination of Mayer’s haematoxylin, alcian blue (pH 2.5) and picrosirius red (5 min, 15 min and 1 h respectively with dH_2_O washes between) prior to imaging using a 3D Histech Pannoramic P250 slide scanner.

### 2.10. Orientation analysis

Alignment of collagen and cells within the AF regions of the bioprinted constructs was investigated using the OrientationJ plugin for ImageJ. AFM and phalloidin images across the entire radius of the AF regions were utilised to assess alignment of collagen and cells as a consequence of any collagen alignment caused by the printing process. Orientation analyses were conducted on 14 images of each region to pseudo-colour raw files for pixel orientation and the ‘dominant direction’ function was used to quantify coherency with a higher number indicating increased levels of alignment. To establish the effect of bioprinting on collagen and cell alignment samples were compared to controls in the form of unprinted, mould casted, cell-laden collagen hydrogels reticulated for 40 min at 37 °C in 24-well plates.

### 2.11. Statistical analyses

All experiments were conducted with *N* = 3 constructs per analysis. All quantitative data presented was analysed for statistical significances using a one-way ANOVA with post-hoc t tests. A *p*-value of less than 0.05 was considered to represent a significant difference.

## 3. Results

### 3.1. Generation of an IVD analogue with structural and mechanical anisotropy

**Figure 2.**
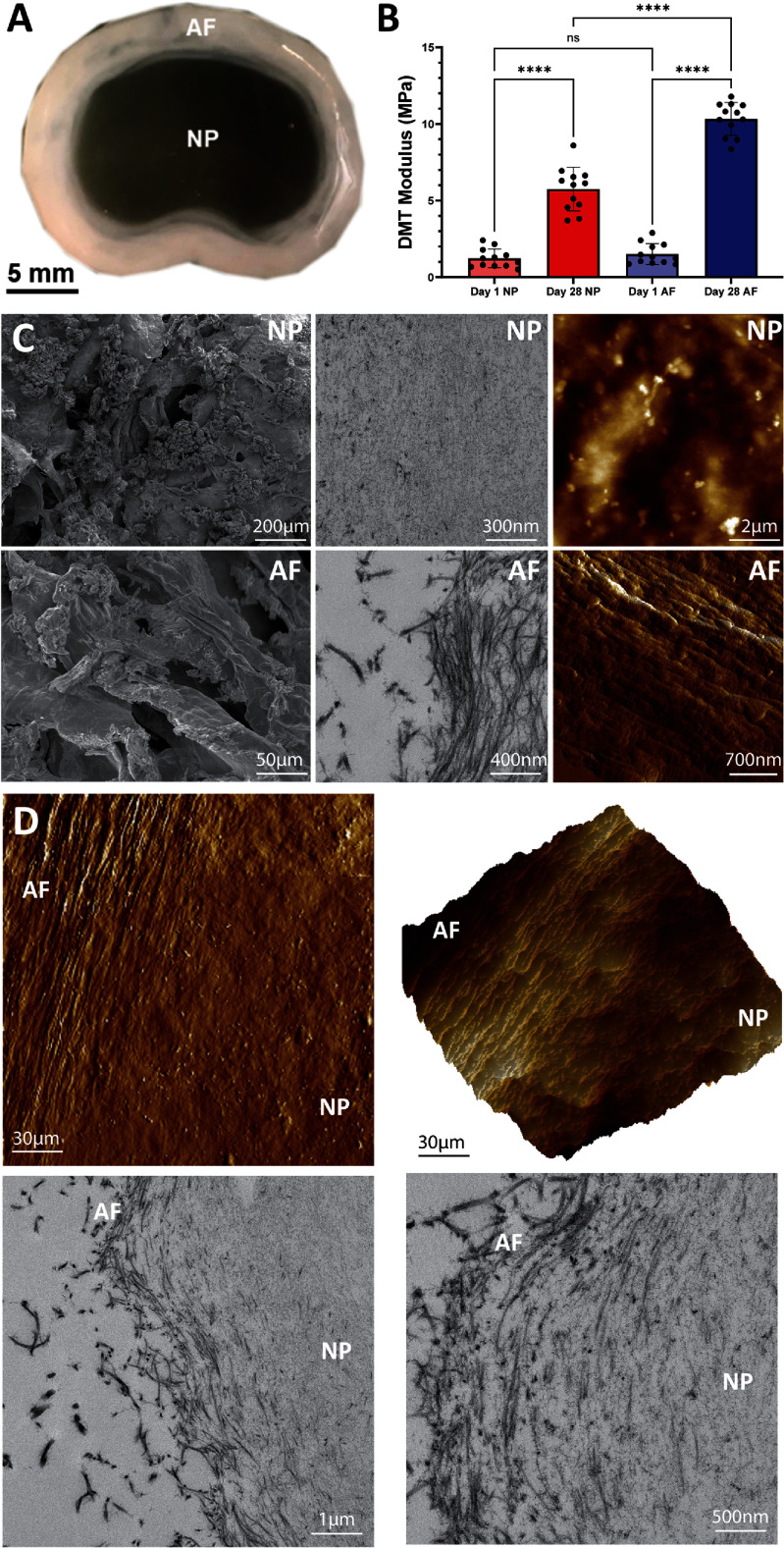
Mechanical and structural characterisation of SLAM bioprinted IVD analogues. (A) Photographic image of a printed construct showing clearly defined NP and AF regions. (B) DMT modulus of the NP and AF regions at days 1 and 28 post-printing. *N* = 3; ****P < 0.0001; one-way ANOVA. (C) SEM, TEM and AFM of the NP and AF regions. (D) AFM and TEM of the NP-AF interface.

SLAM bioprinting of gellan gum and collagen facilitated the generation of constructs that matched the gross morphology of the native IVD tissue (figure 2(A)). Mechanical mapping with AFM revealed no significant differences in stiffness between NP and AF regions (DMT moduli: NP = 1.2 MPa; AF = 1.5 MPa). However, by day 28 both regions had increased in stiffness (DMT moduli: NP = 5.8 MPa; AF = 10.3 MPa) and a clear regional difference was observable, with the AF region significantly stiffer than the NP (*P* < 0.0001).

SEM, TEM and AFM analysis of the two construct regions (NP and AF) revealed structural differences (figure 2(C)) with an amorphous structure in the polysaccharide-rich matrix in the NP and an AF comprised of collagen fibres with evidence of alignment. Moreover, TEM and AFM analysis of the interface between the two zones highlighted the presence of a highly defined gradient in matrix structure with aligned collagen fibres transitioning and dissipating into the gellan-rich NP (figure 2(D)), reminiscent of the native IVD [29].

### 3.2. Recapitulating key biological features of IVD tissue

**Figure 3.**
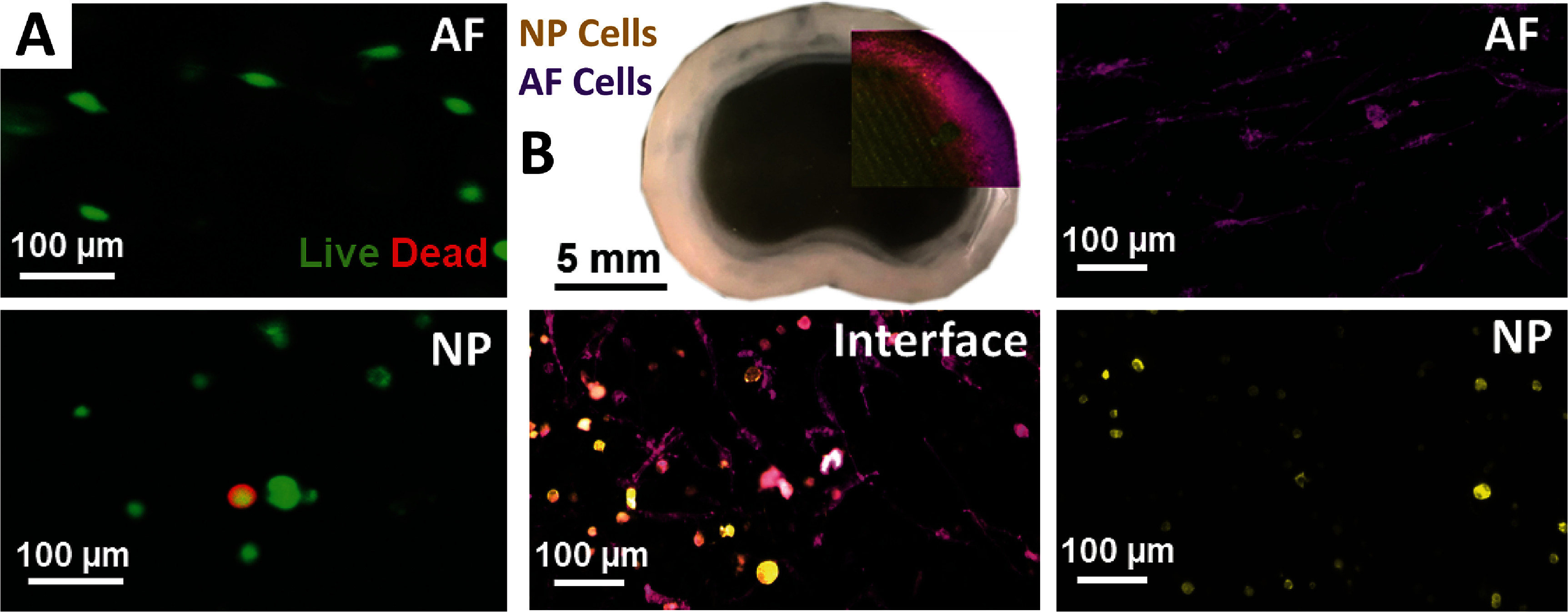
Cell viability and tracking of IVD cells in bioprinted constructs. (A) Representative Live/Dead stain of human NP and AF cells within a bioprinted construct 24 h post-printing. (B) Whole bioprinted IVD showing partial overlay of printed cells labelled with cell tracker dyes. NP cells pseudo-coloured yellow, AF cells pseudo-coloured purple. Higher magnification images of NP and AF cells within each region and a combination of the two cell types with distinct morphologies at the interface. Staining performed on the constructs 28 d post-printing.

Analysis of NP and AF cells within the bioprinted IVD constructs revealed evidence of a capacity to stimulate region-specific morphological responses. This biological anisotropy was generated as a result of printing the two regions of the construct containing two different materials without compromising cell viability (figure 3(A)). Within the AF region of the construct AF cells adopted high levels of cell elongation, while NP cells in the central NP region adopted a rounded morphology, with a mix of phenotypes observable at the interface of the two regions (figure 3(B)).

**Figure 4.**
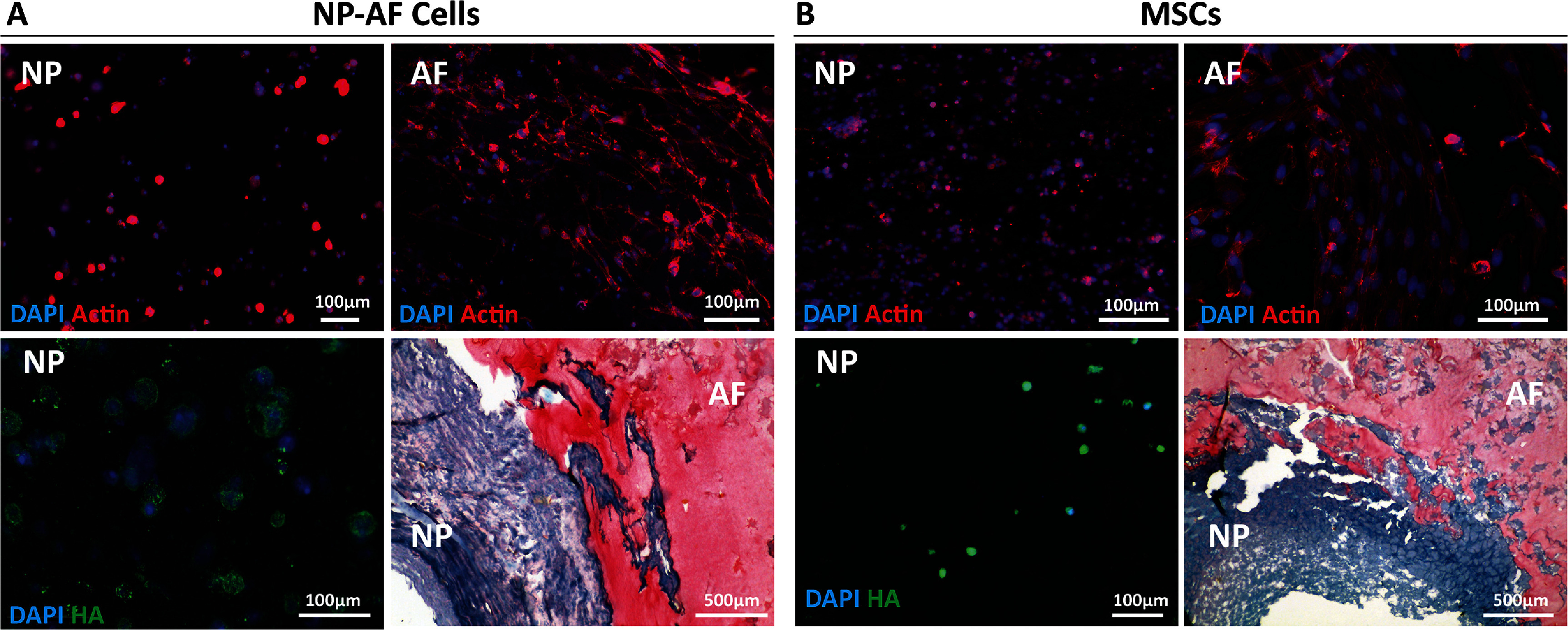
Biological characterisation of SLAM bioprinted IVD analogues. (A) Representative actin and hyaluronan (HA) staining of NP and AF cells in a bioprinted construct 28 d post-printing showing regional morphology differences and hyaluronan expression by NP cells. Representative alcian blue-picrosirius red histology demonstrating integration of NP cell and AF cell-seeded NP and AF regions 28 d post-printing. (B) Representative actin and hyaluronan (HA) staining of MSCs in a bioprinted construct 28 d post-printing showing regional morphology differences and hyaluronan expression by NP cells. Representative alcian blue-picrosirius red histology demonstrating integration of MSC-seeded NP and AF regions 28 d post-printing.

Actin staining of NP and AF cells confirmed regional differences in morphology, with rounded NP cells in the NP region and elongated cells in the AF region (figure 4(A)). NP cells also demonstrated expression of hyaluronan, providing evidence that NP cells were depositing new ECM components into the surrounding matrix. Histological analysis further highlighted the ability to embed cells into a structured tissue gradient, with the polysaccharide-rich NP staining positively with alcian blue and the collagenous AF staining positively with picrosirius red. Crucially, the histological analysis also further evidenced the presence of a defined tissue interface between the NP and AF regions with the integration of a both proteoglycan and collagen-rich matrices linking the two construct areas together.

Interestingly, when constructs were printed to contain MSCs in both regions, clear morphological differences were again apparent, with rounded cells in the NP region and elongated, aligned cells in the AF region (figure 4(B)). Like native NP cells, MSCs in the NP region of the construct were also found to be depositing cell-secreted hyaluronan suggesting a transition from multipotency into a more NP-like phenotype. Tissue morphology again demonstrated a clearly defined interface between the two regions.

### 3.3. Regulation of collagen and cell alignment with bioprinting

**Figure 5.**
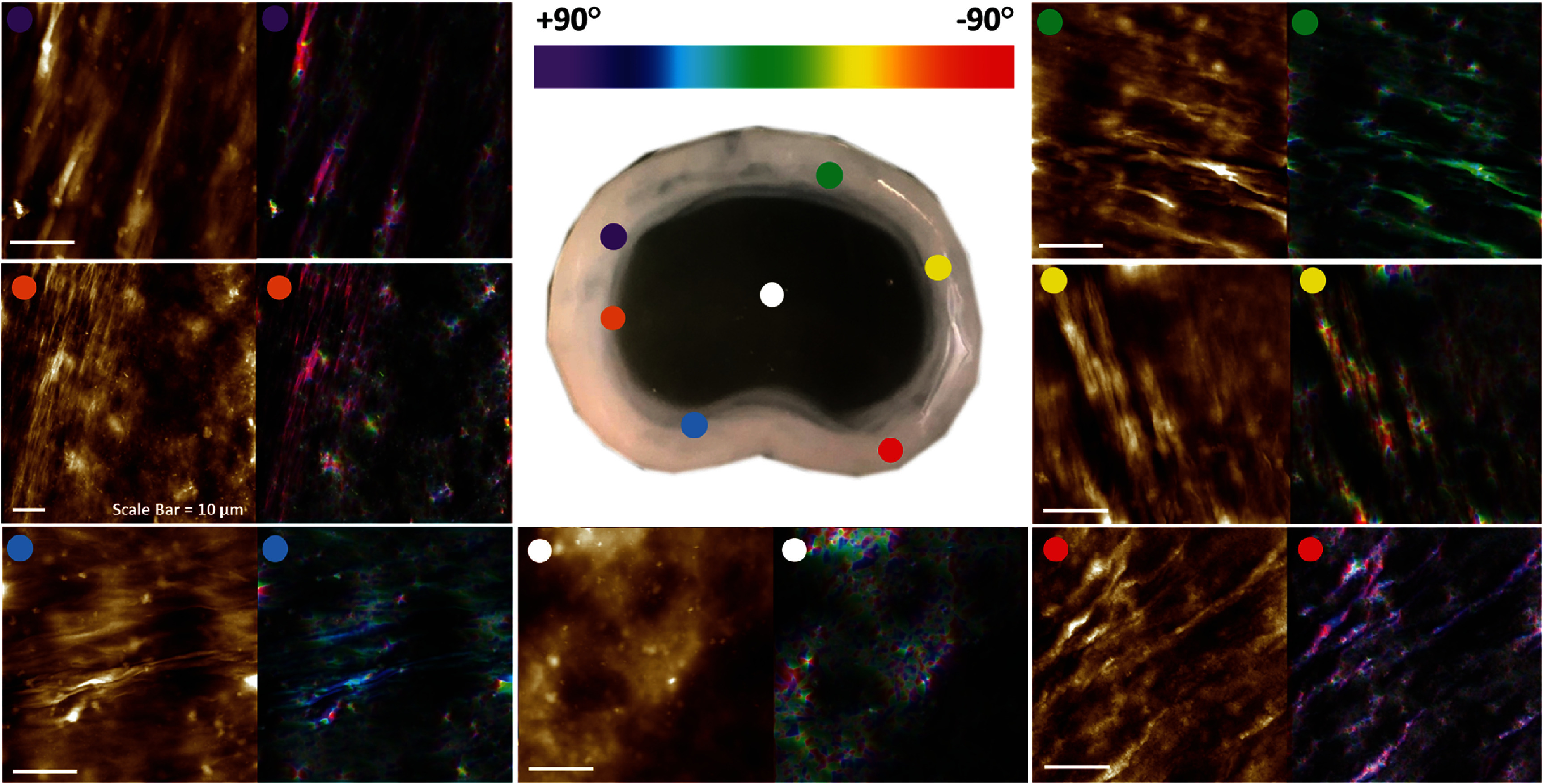
Alignment of collagen in the AF of SLAM bioprinted IVD analogues. AFM and pseudo-coloured AFM images, created using OrientationJ within ImageJ, demonstrating collagen alignment around the AF circumference. No orientation could be identified within the NP region (white dot).

AFM mapping around the circumference of the AF region of the bioprinted constructs revealed the presence of highly aligned collagen fibres that appeared to mirror the circumferential annular ring nature of the native AF ECM (figure 5).

**Figure 6.**
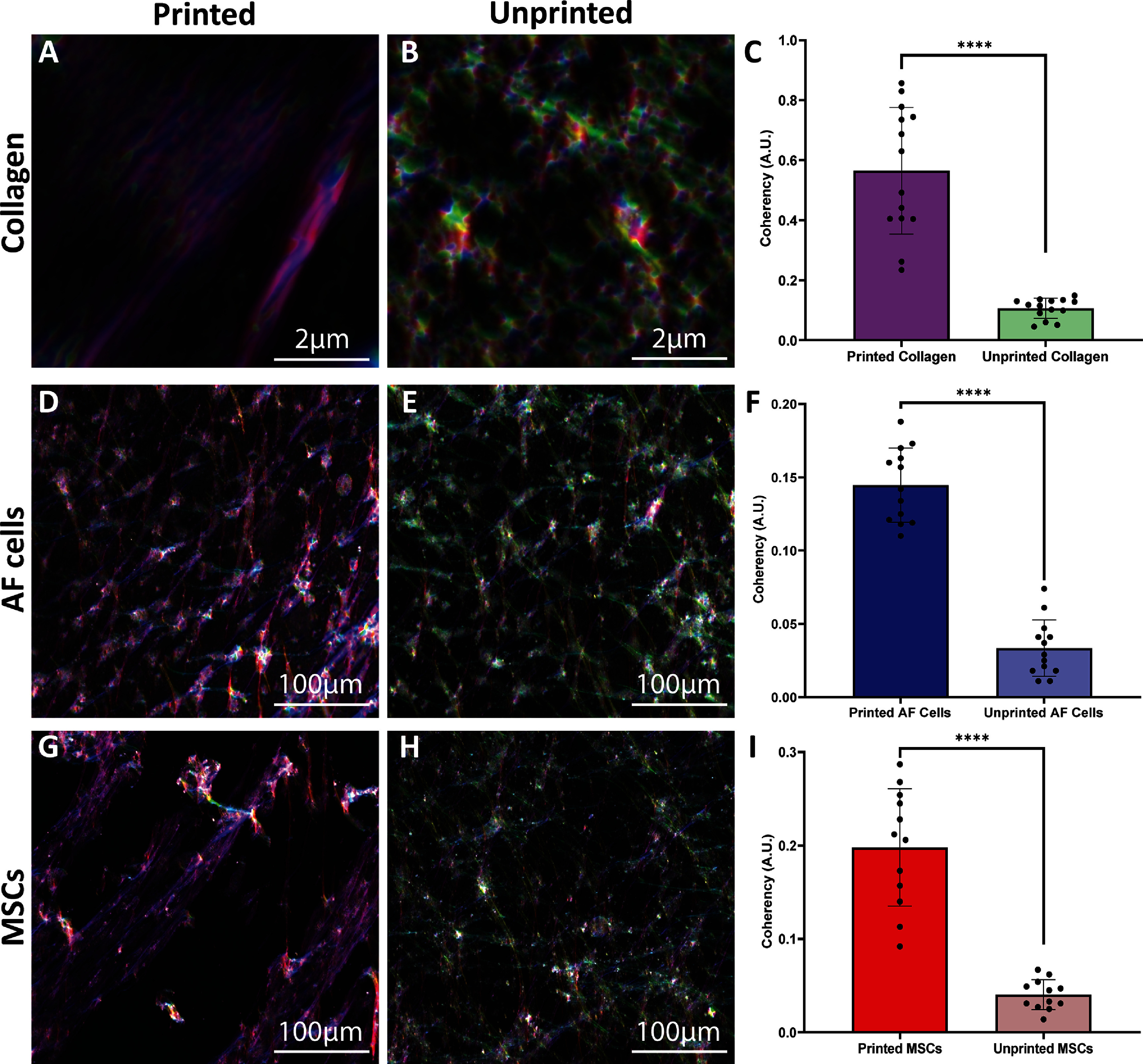
Collagen and cell alignment is triggered via bioprinting. (A)–(C) Pseudo-coloured images and corresponding coherency analysis for collagen in bioprinted IVD constructs and unprinted collagen gels. (D)–(F) Pseudo-coloured images and corresponding coherency analyses for AF cells in bioprinted IVD constructs and unprinted collagen gels. (G)–(I) Pseudo-coloured images and corresponding coherency analyses for MSCs in bioprinted IVD constructs and unprinted collagen gels. *N* = 3; ****P < 0.0001; one-way ANOVA. A.U. = arbitrary units.

Coherency analysis using OrientationJ demonstrated that this alignment was a direct result of bioprinting the collagen in the AF, as unprinted (mould casted) collagen exhibited significantly lower coherency values and, instead, represented a more random fibrous network (figure 6(A)). Embedded AF cells (figure 6(B)) and MSCs (figure 6(C)) within bioprinted AF regions also exhibited a high level of cell alignment, while the same cells embedded in unprinted collagen formed more random cell networks with significantly lower coherency values.

## 4. Discussion

This study explored the potential of generating IVD analogues using SLAM bioprinting. By applying this advanced manufacturing technique, key structural and biological elements of the IVD were recreated. Models such as this can provide valuable tools for probing and understanding the biological mechanism behind IVD tissue formation and degeneration whilst simultaneously reducing the need for animal models.

SLAM bioprinting facilitated the generation of IVD analogues that recapitulated structural, morphological and biological components present in the native tissue. Mechanical mapping following gelation revealed no significant difference in stiffness between NP and AF regions, but notable regional differences in gel morphology. Samples were incubated in culture medium throughout gelation which triggered an anionic gelation mechanism in the gellan-rich NP. It is well documented that anionic gelation of gellan gum with cell culture media results in a soft gel as monovalent ions do not directly crosslink the polymer structure [30–32]. Instead, they suppress the influence of counterions on the polymer chain and encourage gelation by intermolecular interactions; a ‘stiffer’ gel can be generated with divalent ions that covalently link the polymer chains. In contrast, while the stiffness of the AF region was not significantly different to the NP, the collagen present in the AF was reticulated under conditions that resulted in the generation of a network of highly aligned collagen fibres.

Interestingly, both regions exhibited a significant increase in stiffness over the 28 day culture period (figure 2(B)), with the AF region becoming significantly stiffer than the NP region. It is likely that cells within both regions of the bioprinted construct were actively depositing matrix, which contributed to the overall increased stiffness and the matrix-reinforcement effect of cells depositing materials like hyaluronan into polysaccharide gels has previously been observed [33, 34]. Alignment of collagen has also been repeatedly demonstrated to increase matrix stiffness [35, 36] and cells embedded within collagenous *in vitro* matrices have the capacity to remodel and increase its stiffness [37]. This could further explain the increased matrix stiffness in the collagenous AF region of the construct compared to the NP region.

The bioprinted constructs also exhibited structural properties that were highly analogous to native IVD tissue [38]. SEM, TEM and AFM of the NP demonstrated the presence of an amorphous matrix with a typical morphology of a polysaccharide network. This closely matches what is commonly observed in the NP region of human IVD tissue. More interestingly, the AF regions of the construct contained networks of highly aligned collagen fibres (figures 2(C) and 4). This alignment appeared to mirror the annular nature of native AF tissue (figure 4), suggesting that the process of printing the collagen exhibited control over the structuring of the matrix, favouring the formation of aligned over random fibre networks (figure 5). During the bioprinting process the collagen is ejected through a circular nozzle with an inner diameter of 300 *μ*m. The shear and extensional forces present within the nozzle creates an osmotic pressure that may force the collagen molecules into closer proximity, encouraging intermolecular interactions resulting in alignment [39]. Furthermore, a previous study [25] has demonstrated that SLAM bioprinting using the same platform as utilised here yields filaments that can match this resolution (200 *μ*m in diameter). Printing collagen filaments at such a resolution could thus stimulate alignment along the length of the filament in order to facilitate the formation of elongated, mature fibres. AFM, TEM and SEM supports this theory with evidence of the formation of intertwined, rope-like bundles of collagen that are often markers of more mature fibres (figures 2(C) and 5), although more work is required to determine the potential influence of other factors such as base-polymer concentration, or interaction with support bath, that may influence collagen alignment.

This organisational alignment of collagen in the AF is highly analogous to native AF tissue and the ability to interface this with a polysaccharide-rich NP (figure 2(D)) is a development of considerable significance that has not been previously reported in the field [5, 6, 15, 29]. The interfacing of the two materials is further evidenced by the AFM and histological data which clearly highlights the presence of a collagen-rich AF, a polysaccharide-rich NP, an interface between the two and a structural and mechanical gradient transition from NP-AF which is commonly observed in IVD tissue [7] (figure 3(D)). Together, these data highlight the ability to generate a structure that can mechanically and structurally reflect human IVD tissue.

The bioprinting process also facilitated the recapitulation of key biological properties of IVD tissue (figure 3). Cells within the NP region adopted and retained a rounded, chondrocyte-like morphology as seen in native tissue (figures 3 and 4). Moreover, the cells were found to be depositing hyaluronan, a key matrix component in NP tissue [40, 41]. More importantly, in MSC loaded constructs, the same phenomenon was observed with rounded cells depositing HA into the surrounding matrix. This suggests that initiation of differentiation into a more chondrogenic/NP-like phenotype within the NP was triggered, demonstrating the potential to study IVD tissue formation within this model.

In the AF, both AF cells and MSCs were observed to adopt an elongated, aligned morphology similar to observations made in native AF tissue. Alignment of AF cells is a key biological feature of the IVD and this was successfully recreated. However, in the case of MSCs, this controlled alignment is a critical development for two reasons. Firstly, it provides a potential platform for studying AF formation where multipotent cells transition into a highly aligned, AF cell phenotype as the tissue matures. Secondly, it demonstrates the potential to control the morphology and alignment of stem cells by embedding within an aligned, bioprinted collagen matrix; a mechanism which carries applications beyond IVD biology.

A cellular interface could be observed between the two morphologies, but this could be controlled by designing the bioprinting G-code such that the NP and AF still contained segregated regions of the two cell types which is a critical factor to replicate for modelling IVD tissue. The cellular alignment with the AF regions of the constructs was also observed to be a direct result of the alignment of collagen fibres as this was only observed when cells were exposed to bioprinted, aligned collagen in the AF and not seen in random, unprinted collagenous networks.

These data therefore represent a platform for creating IVD tissues highly analogous to native tissues. This could act as a platform for probing the poorly understood mechanisms of human IVD tissue formation by providing an *in vitro* environment that is a close representation of the native tissue environment. Moreover, this study has demonstrated the ability to simultaneously control the cell morphology of multiple cell types by directing the structuring of 3D cell culture matrices. The potential to control IVD cell morphology and phenotype is valuable to the field of IVD research. However, the capacity to exhibit this same level of control over MSC morphology and phenotype is arguably of even greater impact. Exhibiting control over stem cell differentiation via regulation of extracellular environments provides a tool for replicating many of the mechanisms by which stem cell behaviour is regulated in tissue formation.

Furthermore, the use of SLAM bioprinting is particularly significant as it allows the precise structuring of low viscosity materials such as gellan and collagen, which is challenging to achieve with conventional techniques. This method enables the creation of complex structures with defined interfaces and aligned collagen fibres, mimicking the native architecture of IVD tissues. From a clinical perspective, this platform has the potential to contribute to advancing research translation by enhancing understanding of IVD biology and providing more reflective *in vitro* models for improved clinical translation.

The mechanism outlined in this study can, however, also be applied to applications beyond IVD tissue wherever control over stem cell morphology and phenotype is crucial. With the right level of translation, this technology can be applied to fields where the precise control of cell behaviour is paramount to progressing towards new therapeutic discoveries.

## 5. Conclusion

This study has successfully employed SLAM to fabricate IVD analogues with accurate AF architecture and a NP-AF interface, addressing the need for translatable *in vitro* models for disc disease research. By utilising a biphasic hydrogel construct consisting of gellan gum and type I collagen to mimic NP and AF regions respectively, this model not only recapitulated the microstructural characteristics of these regions but also facilitated the incorporation of NP and AF cell lines. In native tissue, the NP is populated by rounded NP cells and the AF is solely comprised of elongated, aligned AF cells and this could be replicated in the bioprinted construct. Furthermore, the same effects could be triggered when IVD cells were replaced with human MSCs, demonstrating the potential to use SLAM bioprinting to control stem cell phenotype and morphology by regulating the structuring of the extracellular environment. The established SLAM-based IVD analogue holds promise not only for investigating IVD-like tissue formation mechanisms, but also for extending its applications beyond IVD biology to broader tissue engineering and regenerative medicine endeavours.

## Data Availability

All data that support the findings of this study are included within the article (and any supplementary files).
